# Reproducibility of and sex differences in common orthopaedic ankle and foot tests in runners

**DOI:** 10.1186/1471-2474-15-171

**Published:** 2014-05-23

**Authors:** Maarten P van der Worp, Anton de Wijer, J Bart Staal, Maria WG Nijhuis- van der Sanden

**Affiliations:** 1 Department of Physical Therapy, Academic Institute, University of Applied Sciences Utrecht, Utrecht, the Netherlands; 2 Institute Health Studies, HAN, University of Applied Sciences Nijmegen, Nijmegen, the Netherlands; 3 Scientific Institute for Quality of Healthcare, Radboud university medical center, Nijmegen, the Netherlands; 4 Department of Oral Function & Prosthetic Dentistry, Radboud university medical center, Nijmegen, the Netherlands; 5 Department of Rehabilitation, Radboud university medical center, Nijmegen, the Netherlands

**Keywords:** Injury prediction, Evaluation, Reliability, Agreement, Running

## Abstract

**Background:**

For future etiologic cohort studies in runners it is important to identify whether (hyper)pronation of the foot, decreased ankle joint dorsiflexion (AJD) and the degree of the extension of the first Metatarsophalangeal joint (MTP1) are risk factors for running injuries and to determine possible sex differences.

These parameters are frequently determined with the navicular drop test (NDT) Stance and Single Limb-Stance, the Ankle Joint Dorsiflexion-test, and the extension MTP1-test in a healthy population. The aim of this clinimetric study was to determine the reproducibility of these three orthopaedic tests in runners, using minimal equipment in order to make them applicable in large cohort studies. Furthermore, we aimed to determine possible sex differences of these tests.

**Methods:**

The three orthopaedic tests were administered by two sports physiotherapists in a group of 42 (22 male and 20 female) recreational runners. The intra-class correlation (ICC) for interrater and intrarater reliability and the standard error of measurement (SEM) were calculated. Bland and Altman plots were used to determine the 95% limits of agreements (LOAs). Furthermore, the difference between female and male runners was determined.

**Results:**

The ICC’s of the NDT were in the range of 0.37 to 0.45, with a SEM in the range of 2.5 to 5 mm. The AJD-test had an ICC of 0.88 and 0.86 (SEM 2.4° and 8.7°), with a 95% LOA of -6.0° to 6.3° and -5.3° to 7.9°, and the MTP1-test had an ICC of 0.42 and 0.62 (SEM 34.4° and 9.9°), with a 95% LOA of -30.9° to 20.7° and -20° to 17.8° for the interrater and intrarater reproducibility, respectively.

Females had a significantly (p < 0.05) lower navicular drop score and higher range of motion in extension of the MTP1, but no sex differences were found for ankle dorsiflexion (p ≥ 0.05).

**Conclusion:**

The reproducibility for the AJD test in runners is good, whereas that of the NDT and extension MTP1 was moderate or low. We found a difference in NDT and MTP1 mobility between female and male runners, however this needs to be established in a larger study with more reliable test procedures.

## Background

Running has become popular in the last decades [1]. The Royal Dutch Athletics Federation (KNAU) has estimated that about 12.5% of the Dutch population runs regularly, and that the popularity of running events is still growing [2]. Running is an inexpensive form of vigorous-intensive physical activity and can be done anywhere and at any time. It is also a basic aspect of many recreational and professional sports. However, running can cause injuries, especially of the lower extremities, with incidences varying between 20% and 79% and with the knee as most common site of injury followed by lower leg and foot [3]. Knowledge of potential risk factors is needed in order to prevent running injuries [4]. The exact causes of running injuries remain to be determined, but are likely to be diverse [3].

For future etiologic cohort studies of runners it is important to identify whether (hyper)pronation of the foot, decreased ankle joint dorsiflexion (AJD) and the degree of the extension of the MTP1 are risk factors for running injuries. To measure the extent of foot pronation, AJD and the extension of the MTP1, reproducible orthopedic tests are essential.

Bennett et al. [5] and Buist et al. [6] found in their prospective studies a positive navicular drop test (NDT > 10 mm) as predictor for running related injuries. In the same study of Buist et al. [6] and in the case–control study of Van Mechelen [7] dorsiflexion was not found as risk factor and no difference was found in ankle joint mobility between injured and non-injured runners. Further prospective studies are needed to include/exclude the ankle range of motion as possible risk factor for running injuries. Also the extension of the MTP1 is, by our knowledge, not included in etiology studies as a risk factor for running injuries and needs future research.

To determine the extent of ND, AJD and extension of the MTP1, the navicular drop test (NDT), a method to classify the degree of foot pronation, the weight bearing AJD-test and the MTP1-test are used. The NDT is moderately reliable [8,9]. In the study of Vinicombe et al. [8], five clinicians performed the NDT twice in 20 healthy participants (13 women and 7 men, mean age 20 ± 2 years), with an ICC ranging from 0.33 to 0.76, with a 95% confidence interval of 1.5 mm to 3.5 mm. Shultz et al. [9], in a study of the reliability of measurements of lower extremity anatomical characteristics, reported the intrarater and interrater reliability of the NDT to be 0.91–0.97 and 0.56- 0.76, respectively.

Measurement of ankle joint dorsiflexion (AJD) with an inclinometer and extension of the MTP1 in a weight-bearing position with a goniometer proved to be reliable orthopaedic tests [10,11]. Munteanu et al. [11] found measurements of the AJD, with an inclinometer and in a weight- bearing position with the knee extended, to have a high intra- and interrater reliability (>0.77 and > 0.90, respectively) in 30 asymptomatic participants.

Hopson et al. [10] found an ICC of 0.98 for the reliability of the MPT1 extension test in static weight-bearing position when measured in 10 women and 10 men aged 21– 43 years.

However, there are no data in the literature on the reliability of these orthopaedic tests in healthy adult runners, a population of particular interest for screening purposes. Clinical measurements of the ND, AJD and extension of the MTP1 can be used to guide decisions regarding preventive treatment strategies in runners, including the use of orthotics and modification of footwear.

In conclusion, above mentioned studies focused on reliability of the NDT, AJD-test and extension MTP1-test in healthy adults. However, these tests seem to be important to identify runners with higher injury risk and for prevention purpose. Moreover, in this study, the protocols in the literature of the NDT [8], AJD-test [11] and extension MTP1-test [10] were adapted for the use in our planned prospective cohort study of female runners (n = 433). This adaptation was necessary for practical reasons, which required that these orthopaedic tests are performed in maximal 10 minutes, on location and with a minimum of measurement tools and equipment.

For the NDT [8], in our protocol a ruler was used instead of a blank card [12] and the sitting position was used as neutral position of the foot instead of palpating the talar head [13], so the NDT could be determined directly and the measurement time was minimized. The performing times of the protocols of the AJD-test [11] and the extension of MTP1-test [10] were optimized by refraining from using a tapeline and standardized step length, but extra attention was paid to maximal stretch of the posterior leg and MTP joint, respectively. Consequently, by deviating of existing protocols, the agreement (as a characteristic of the protocol and measurement instrument itself) of these three tests had to be determined as well.

Furthermore, we hypothesized that there is a difference between sexes based on several runner studies [6,14-17] which showed differences in risk profile between male and female runners. This sex difference, regarding the musculoskeletal system, can partly be explained by the difference in NDT, AJD- and MTP1 mobility. In a cohort study of Buist et al. [6] of novice runners, sex-specific risk factors were found: women who had higher values of the NDT were more prone to running related injuries (Hazard ratio 0.85; 95% confidence interval 0.75- 0.97). Although not yet identified as a risk factor, differences in the AJD and the extension of the MTP1 between males and females could also (partially) explain the risk profile difference between males and females. A limited function will change muscle activity and joint loading in the functional chain.

Hence, the aim of this study was to develop and assess the intrarater and interrater reliability and agreement of the NDT, AJD test and extension MTP1 test in weight-bearing position in healthy runners. Secondly, we wanted to compare outcomes of these tests between female and male runners.

## Methods

### Ethics statement

The study was approved by the Medical Ethical Committee of the Radboud University Nijmegen Medical Centre. Written informed consent of the participants was obtained before the study. The participants of the images and videos provided a written consent for the publication of their images and videos.

### Participants

A group of 46 recreational runners (running minimally once a week and minimally 5 km), who were members of a track and field club, running groups, or running on individual basis, was recruited by physiotherapists, trainers, and coaches of the local track and field clubs in Utrecht, the Netherlands. Potential participants were personally invited to participate, were informed about the study and were given the opportunity to volunteer. Runners were eligible if they were 18 years or older, were healthy, and running injury-free at that moment. None of the participants complained of lower extremity pain or spinal pain, and none had medical or neuromusculoskeletal disorders that limited participation in work, sports, or exercise. Forty two runners met the inclusion criteria. All participants provided written informed consent and analyses were performed on anonymous data. The characteristics of the runners who participated in our study are showed in Table 1.

**Table 1:** Demographics of the runners participating in the study

	**Total (n = 42)**	**Males (n = 22)**	**Females (n = 20)**
	**Mean ± SD**	**Mean ± SD**	**Mean ± SD**
Age (yrs)	38.2 ± 12.4	39.1 ± 14.7	37.2 ± 9.3
Weight (kg)	71.3 ± 12.4	80.1 ± 8.3	61.6 ± 8.0
Height (cm)	175.4 ± 8.8	181.8 ± 6.3	168.4 ± 4.9
BMI (kg/m^2^)	23.1 ± 3.0	24.3 ± 2.9	21.7 ± 2.5
Years of running (yrs)	9.8 ± 11.1	10.5 ± 12.6	8.9 ± 9.5
Weekly training frequency (days)	2.4 ± 1.5	2.6 ± 1.9	2.1 ± 0.7
Weekly training distance (km)	22.1 ± 22.2	27.1 ± 28.8	16.6 ± 9.4

SD = Standard Deviation, BMI = Body Mass Index.

### Procedures

The three tests were conducted by two sports physiotherapists (HD and MW) who were specialized in running injuries, board-certified clinical specialists in sports physiotherapy, and members of the International Federation of Sports Physical Therapy (IFSPT). Both examiners attended three 1-hour training sessions prior to data collection, to increase consistency in testing procedure and interpretation.

**Figure 1:**
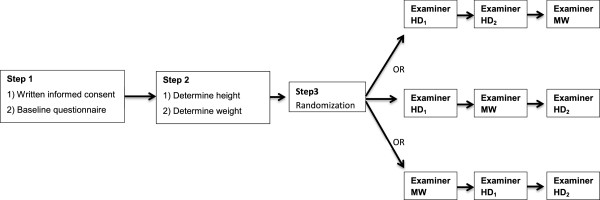
**Flowchart of the procedure of measurements of examiner Maarten van der Worp (MW) and examiner Holger Drechsler (HD); twice; HD**_**1 **_**and HD**_**2**_**.**

After giving written informed consent, each runner completed a baseline questionnaire about his/her running status and injury history. The height and weight were determined. The runners were randomly assigned to the two examiners (MW and HD). To determine intrarater reliability, runners were measured twice by examiner HD (HD_1_ and HD_2_). Figure 1 shows a flow chart of the procedure. Both examiners completed all three static tests once for both legs and feet, randomized in test order by computer, with minimally 10 minutes between measurements of examiner HD. All measurements of one runner were taken on the same day.

### Navicular Drop Test (NDT)

In the current study, a modified version of the navicular drop test described by Vinicombe et al. [8] was used, see Additional file 1: Video 1. In our protocol a ruler was used instead of a blank card [12] and the sitting position was used as neutral position of the foot instead of palpating the talar head [13].

**Figure 2:**
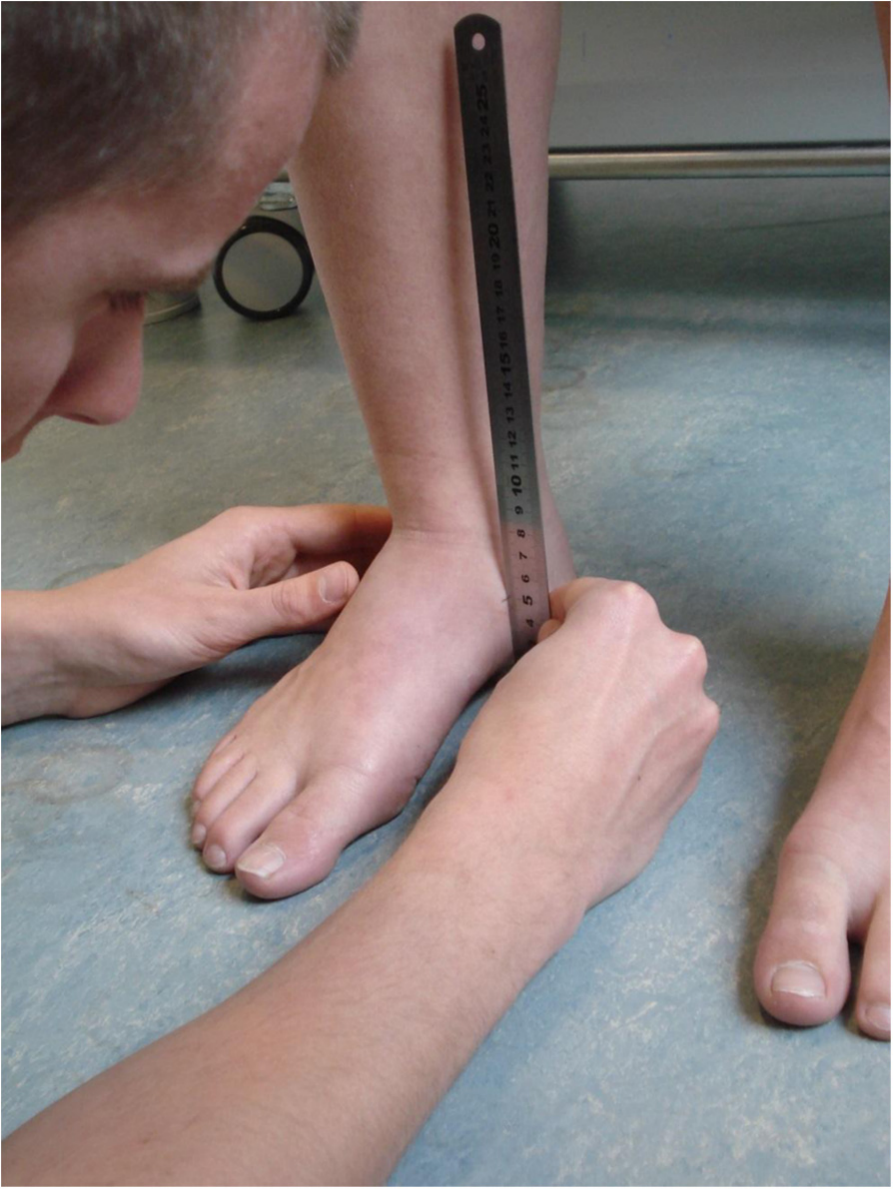
Measuring the height of the medial aspect of the navicular bone in stand position, with a ruler.

The runner was sitting upright with arms crossed in front of the chest, feet flat on the ground and equal weight on both sides, with hip and knees flexed at 90°, and the most medial aspect of the navicular bone was marked. The un-weighted navicular position was the distance from the floor to the point marked on the navicular bone, measured with a ruler. The runner was then asked to stand, without moving the feet, equal weight bearing on both legs and the distance between the navicular marker and the floor was measured again (Figure 2). Then the runner was asked to stand on one leg by flexing the contra-lateral hip and knee 90°, holding a chair for balance and maximum weight bearing on the supporting leg was encouraged. The Single Limb-Stance position was selected because this position reflects the position of the foot during the mid-stance phase of gait [18] and a ruler was used so the navicular drop could directly be determined. The difference between the distance from the navicular marker to the floor in resting position versus standing (NDT; Stance) and resting position versus single limb-stance (NDT; Single Limb-Stance) was scored as the navicular drop standing and navicular drop single limb-stance, respectively [8,19].

### Ankle joint dorsiflexion test

**Figure 3:**
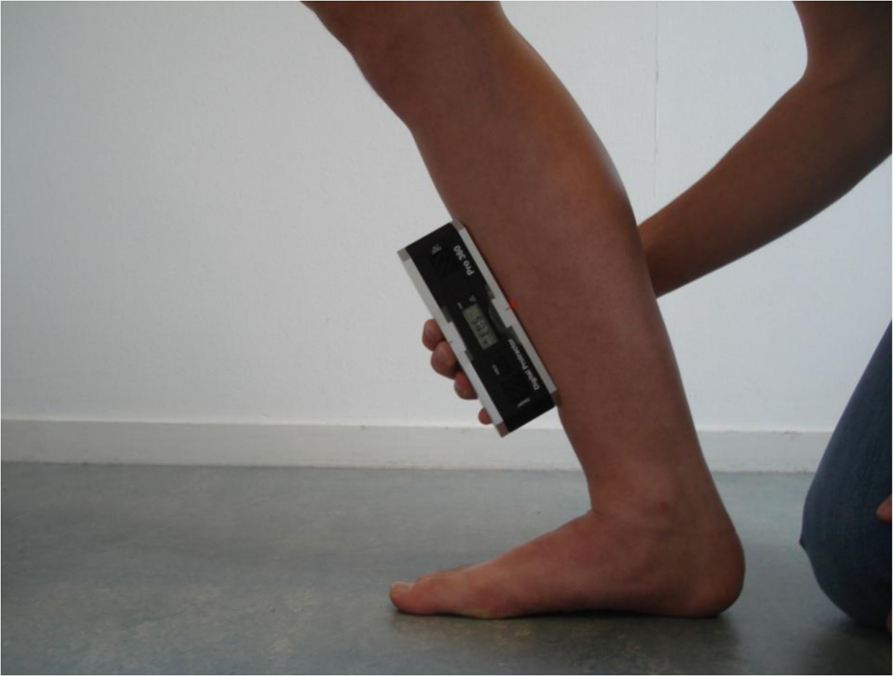
Measurement with an inclinometer of ankle dorsiflexion with extension in the knee.

For measuring the ankle joint dorsiflexion, the protocol described in the study of Munteanu et al. [11] was used, only without using a tapeline, see Additional file 2: Video 2. The runner was asked to step forward with the left leg, so that the right knee was fully extended. The right foot was straight, in line with the left foot. The runner leaned forward until maximum stretch was felt in the right leg while keeping the right knee fully extended and the right heel in contact with the ground; this movement was repeated. If necessary, the runner could put his or her hands on the wall in front, just for keeping balance. The left leg was in a comfortable position to maintain balance and to allow dorsiflexion of the right ankle. The angle between the right tibia and the vertical axis was then measured using a calibrated digital inclinometer (Pro 360 digital protractor; Smart Tool Technology, Inc, Oklahoma City, OK; accuracy = ± 0.1°, maximum resolution = 0.1°). The inclinometer was positioned on a mark made on the mid-part of the anterior side of the tibia between the upper edge of tibial tuberosity and the anterior joint line of the ankle (Figure 3) [11].

### Extension First Metatarsophalangeal Joint test (MTP1-test)

For measuring the mobility of the MTP1 the protocol, as used in the study of Hopson et al. [10], was slightly modified (not standardizing the step length) and used in this study, see Additional file 3: Video 3. With the runner lying on a treatment table, the MTP1 was identified by passive dorsiflexion and plantar flexion of the hallux. Marks were made on the medial aspect of the joint centre and, after palpation, on the medial side of the shaft of the first metatarsal and the proximal phalanx of the hallux. Then the runner was asked to step forwards and to raise the heel of the foot behind, with full extended knee and extending the MTP1 as far as possible while maintaining step length and hallux contact with the floor; the runner could use the wall for balance, if necessary.

Because the knee was fully extended, the maximum elongation of MTP1 reached by instruction and holding balance was no problem, the size of the step length did not affect the outcome of the MTP1 mobility.

**Figure 4:**
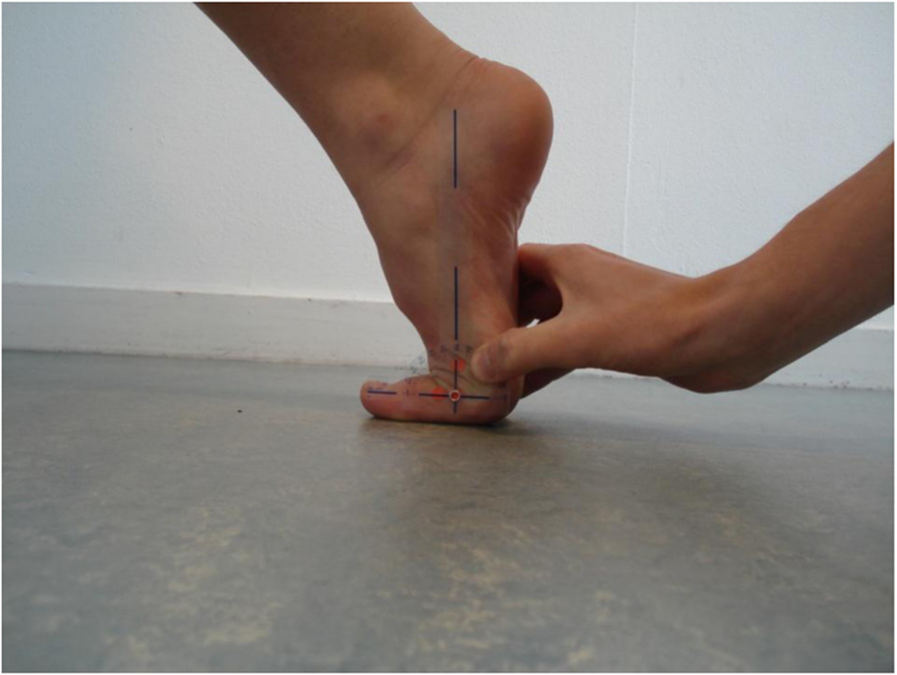
Measurement with a goniometer of the maximal extension of the first metatarsophalangeal joint.

A goniometer (MSD pocket goniometer, baseline 180 degree, transparent plastic) was placed on the skin markers with the centre of the goniometer at the metatarsophalangeal joint, one goniometer-arm line crossing the centre of the mark on the shaft of the first metatarsal and the other goniometer-arm line crossing the centre of the mark of the proximal phalanx of the hallux, respectively (Figure 4).

The value recorded was the maximum MTP1 extension angle while the runner maintained his or her step length position.

### Statistical analysis

The data from the right and left leg and feet were used separately in all calculations. Intraclass correlation coefficients (ICCs) were calculated. ICC model 2.1, with absolute agreement and single measures, was used for intrarater reliability and ICC model 2.2 with absolute agreement and single measures for interrater reliability, respectively [20,21]. The guidelines used for the interpretation of the ICCs were as follows: 0.0 to 0.25 indicated little if any correlation; 0.26 to 0.49 indicated low correlation; 0.50 to 0.69 indicated moderate correlation; 0.70 to 0.89 indicated high correlation; and 0.90 to 1.00 indicated very high correlation [22].

To determine the agreement between the three orthopaedic tests, the standard error of measurement (SEM) and the 95% limits of agreement (LOA) were calculated as measure of ‘total error’ (systematic and random error combined) [23]. SEM “agreement” was calculated for taking in account possible systematic errors [24]. Bland and Altman plots were created by plotting the difference between each measurement and the mean difference of the measurement for the intrarater and interrater agreement, to visualize the possible systematic error and random error of the measurements of one examiner (HD) or the difference between the examiners (HD and MW) [25].

For sex differences comparisons, means, standard deviations, mean differences and 95% confidence interval (CI) of the dependent variables of the three tests were calculated, for which the data of the measurement of examiner MW were used. Independent t-test with an alpha value of 0.05 was used to evaluate sex differences comparisons. Data analysis was performed using SPSS Version 22.0 (SPSS Inc, Chicago, IL).

## Results

Table 2 presents the mean and standard deviation of the measurements of the two examiners (HD twice; HD_1_ and HD_2_), the ICCs with 95% confidence intervals and the SEMs with 95% LOA’s.

**Table 2:** Measurement outcomes of the two examiners MW and HD (twice) and the reproducibility the three orthopaedic tests in all participants (n = 42)

	**HD**_**1**_	**MW**	**HD**_**2**_	**Reliability test**	**ICC (95% CI)**	**S.E.M (95% LOA)**
	**Mean ± SD**	**Mean ± SD**	**Mean ± SD**			
**NDT Stance (mm)**	6.2 ± 2.7	5.8 ± 2.8	6.0 ± 2.8	Interrater	0.45 (0.26- 0.60)	3.2 (-6.1- 5.3)
				Intrarater	0.43 (0.23- 0.59)	2.5 (-5.6- 6.1)
**NDT SL-S (mm)**	5.8 ± 3.1	5.1 ± 2.8	5.8 ± 3.1	Interrater	0.41 (0.21- 0.57)	5.0 (-7.2- 5.8)
				Intrarater	0.37 (0.18- 0.54)	2.5 (-7.0- 6.9)
**AJD test (°)**	48.1 ± 6.6	48.2 ± 6.1	46.8 ± 6.0	Interrater	0.88 (0.82- 0.92)	2.4 (-6.0- 6.3)
				Intrarater	0.86 (0.80- 0.91)	8.7 (-5.3- 7.9)
**MTP I test (°)**	79.4 ± 10.9	74.2 ± 13.1	80.5 ± 10.8	Interrater	0.42 (0.23- 0.59)	34.4 (-30.9- 20.7)
				Intrarater	0.62 (0.47- 0.74)	9.9 (-20.0- 17.8)

HD_1_ = first measurement examiner HD, MW = measurements examiner MW, HD_2_ = second measurements examiner HD, SD = Standard Deviation, NDT = Navicular Drop Test, NDT Stance = Navicular Drop Test Stance, NDT SL-S = Navicular Drop Test Single Limb-Stance, AJD = Ankle Joint Dorsiflexion, MTP I = First Metatarsophalangeal Joint, ICC = Interclass Correlation Coefficients, CI = Confidence Interval, S.E.M. = Standard Error of Measurement, LOA = Limits Of Agreement.

### Navicular Drop Test (NDT)

**Figure 5:**
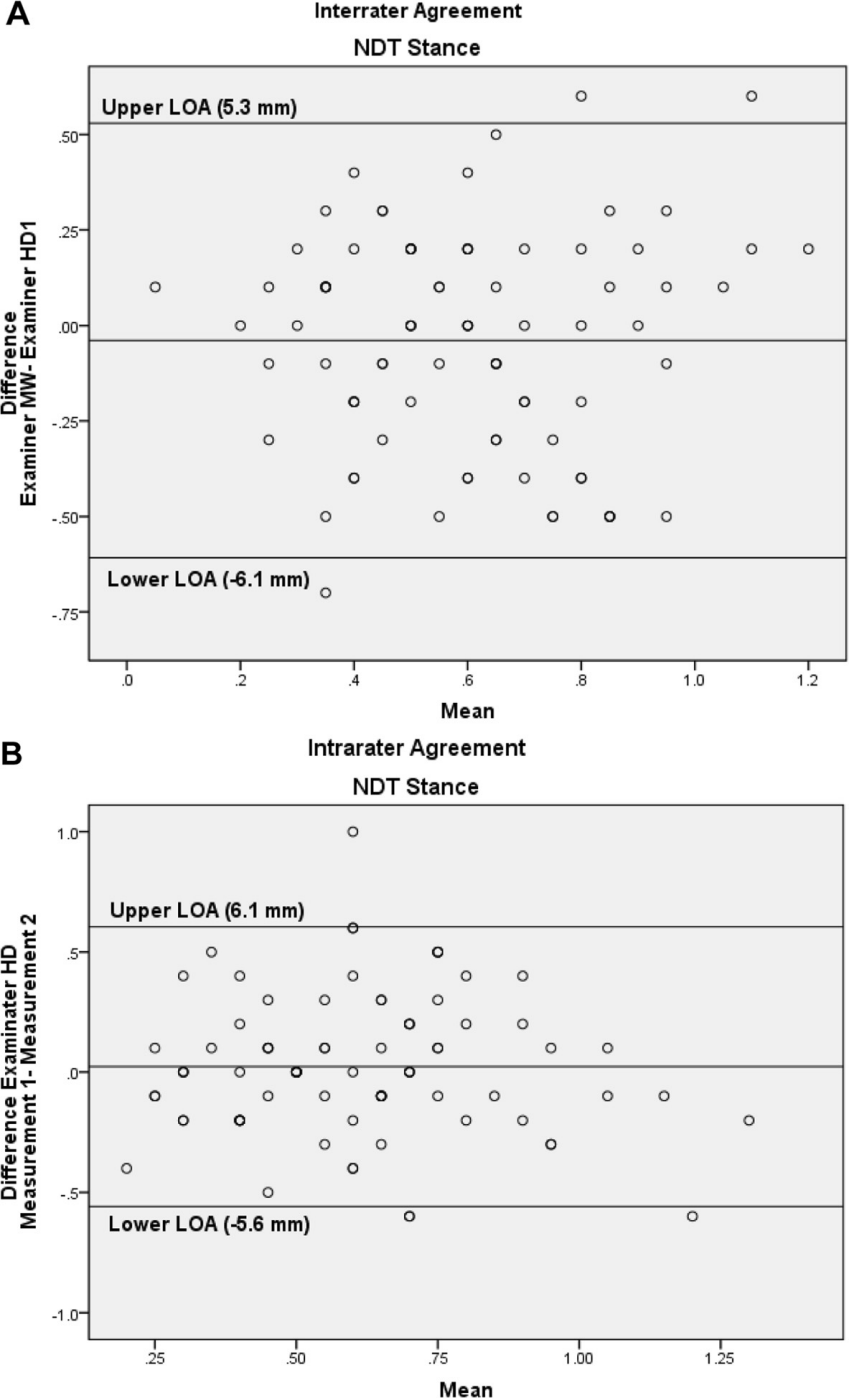
Bland-Altman plots for the interrater (A) and intrarater (B) agreement of the Navicular Drop Test (NDT) Stance, with the 95% limits of agreement (LOA).

*NDT Stance*: Interrater and intrarater ICCs of the NDT Stance measurements were low (ICCs; 0.45 and 0.43, respectively) and with a SEM of 3.2 mm and 2.5 mm respectively. The Bland & Altman plots with the 95% LOAs, Figure 5A and B, for interrater and intrarater reliability respectively, illustrate the low agreement.

**Figure 6:**
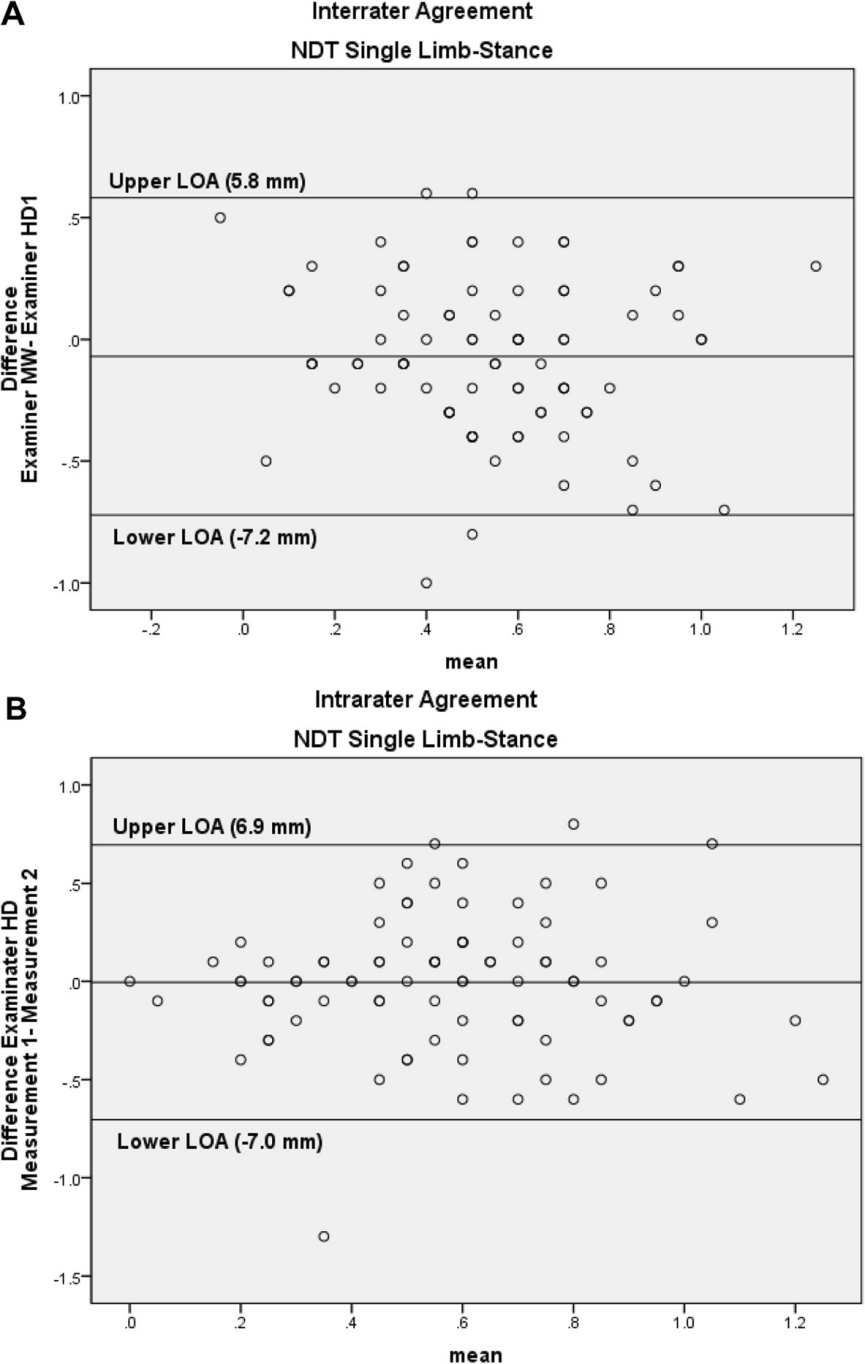
Bland-Altman plots for the interrater (A) and intrarater (B) agreement of the Navicular Drop Test (NDT) Single Limb-Stance, with the 95% limits of agreement (LOA).

*NDT Single Limb-stance*: The ICC of the interrater and intrarater reliability of the NDT Single Limb-Stance was low. The SEMs were 5 mm and 2.5 mm for the interrater en intrarater agreement, respectively. Figure 6A and B shows the Bland & Altman plots with the 95% LOAs of -7.2 to 5.8 mm and -7.0 to 6.9 mm for the interrater and intrarater agreement, respectively.

### Ankle Joint Dorsiflexion Test (AJD-Test)

**Figure 7:**
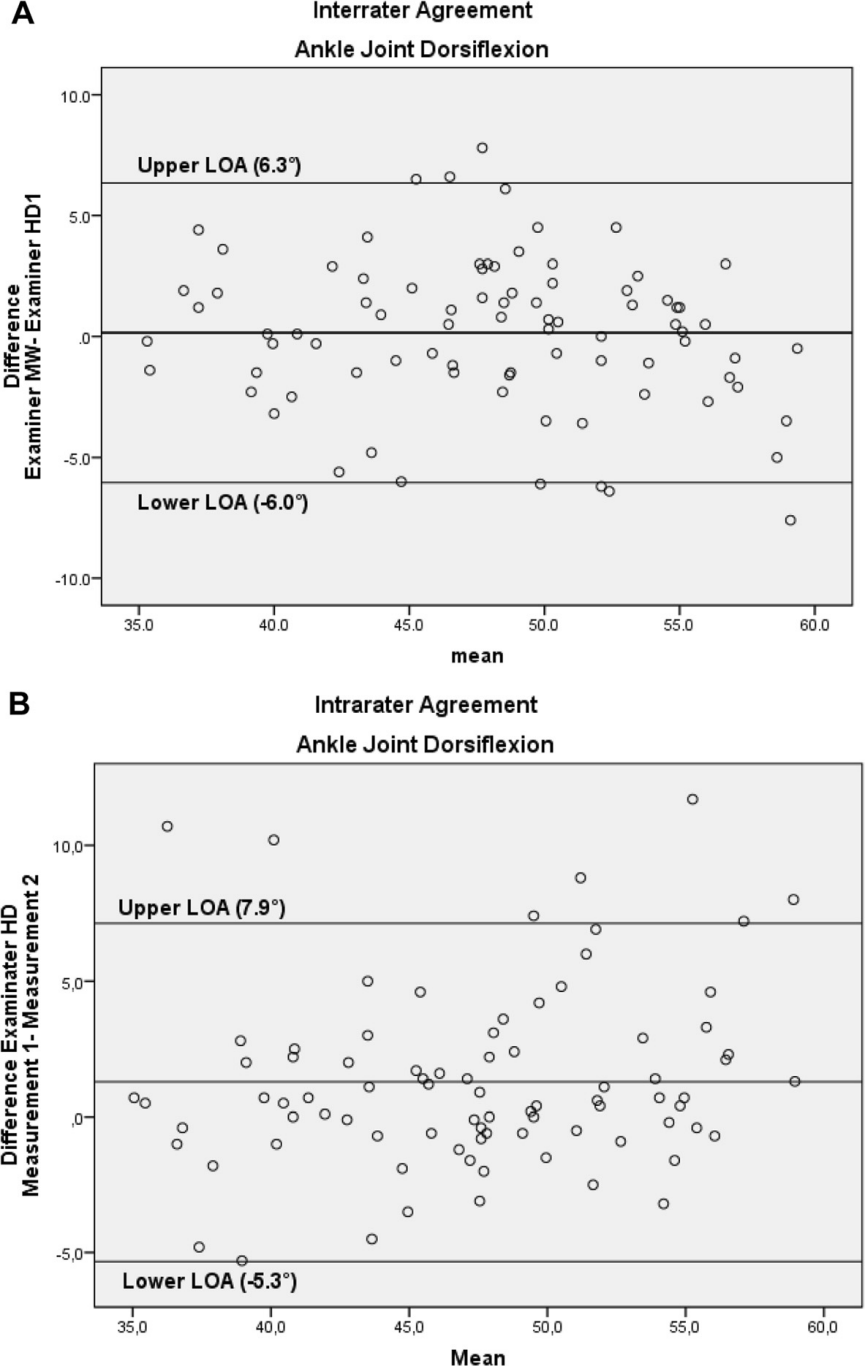
Bland-Altman plots for the interrater (A) and intrarater (B) agreement of the ankle joint dorsiflexion, with the 95% limits of agreement (LOA).

The interrater and intrarater reliability of AJD measurements was high for both (ICCs = 0.88 and 0.86, respectively). The agreement between examiners was lower than within examiners (SEM 2.4° and 8.7°, respectively). The Bland and Altman plots, see Figure 7A and B, reflect the high degree of agreement with 95% LOA’s of -6.0° to 6.3° and -5.3 to 7.9° for the interrater and intrarater agreement, respectively.

### Extension First Metatarsophalangeal Joint Test (MTP1-Test)

**Figure 8:**
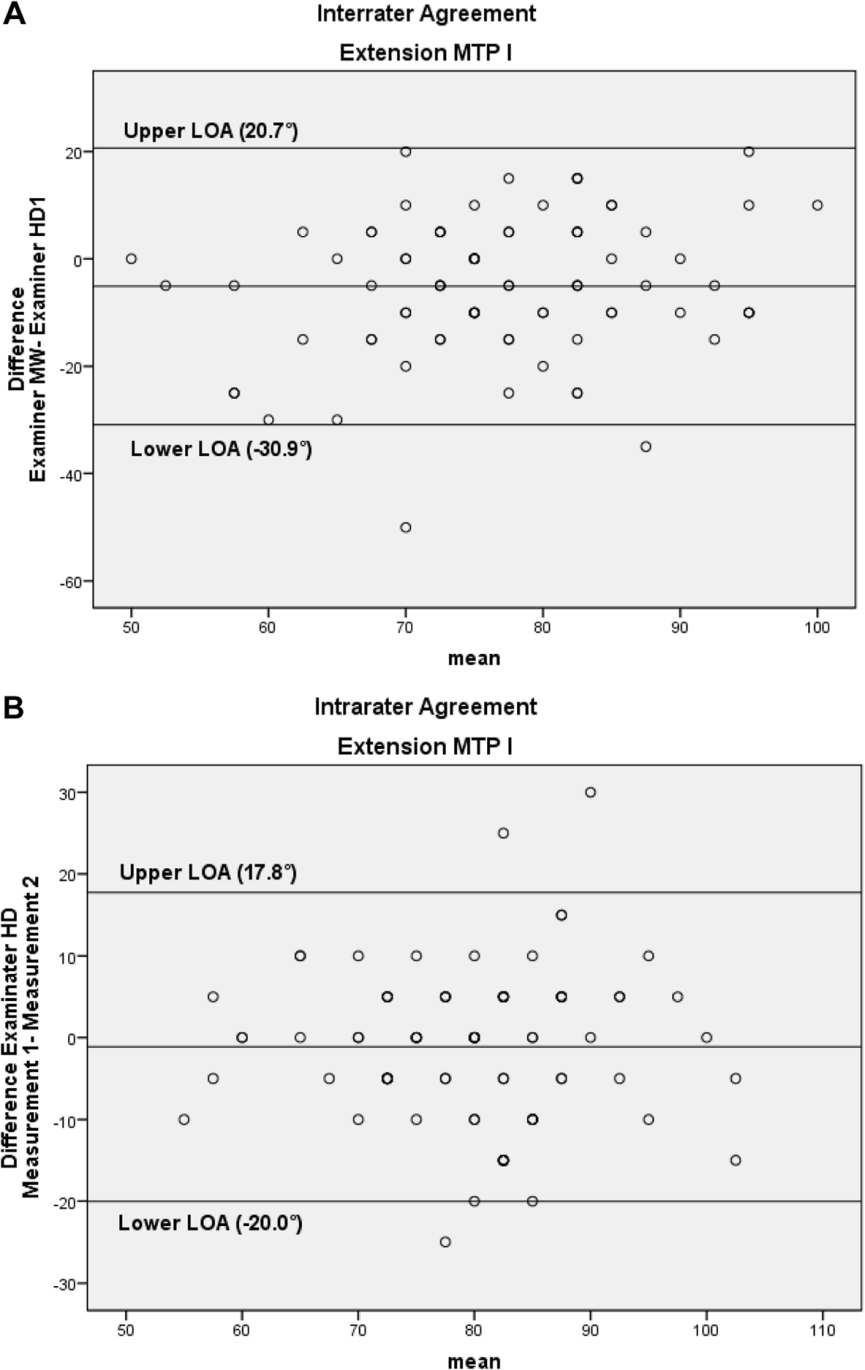
Bland-Altman plots for the interrater (A) and intrarater (B) agreement of the extension of the first metatarsophalangeal joint (MTP1), with the 95% limits of agreement (LOA).

The interrater reliability of the MTP1 test was low (ICC 0.42 and SEM 34.4°) whereas the intrarater reliability was moderate (ICC 0.62; SEM 9.9°). Figure 8A and B, the Bland & Altman plots with the 95% LOAs, illustrates the low and moderate agreement for interrater and intrarater agreement, respectively.

### Sex differences

The outcome measurements of the three orthopaedic tests were described in Table 3. Females demonstrated a significantly lower navicular drop (both for Stance and Single Limb for Stance) with a mean difference of 1.8 mm (p = 0.003) and 1.9 mm (p = 0.002), respectively and a higher extension of MTP1, with a mean difference of 9.1° (p = 0.001). No difference was found in the mobility of the AJD, p-value ≥ 0.05. See Table 3.

**Table 3:** Sex differences of the three orthopaedic tests, with the mean, standard deviation (SD), mean difference, 95% confidence interval and p-values of the measurements outcomes of examiner MW

	**Male (n = 22)**	**Female (n = 20)**			
	**Mean ± SD**	**Mean ± SD**	**Mean difference**	**95% CI**	**P-Value**
**NDT Stance (mm)**	6.7 ± 3.1	4.9 ± 2.1	1.8	0.6- 2.9	0.003^†^
**NDT SL-S (mm)**	6.0 ± 3.0	4.1 ± 2.2	1.9	0.7- 3.1	0.002^†^
**AJD-test (°)**	47.7 ± 5.6	48.9 ± 6.6	1.2	-3.9- 1.4	0.364
**MTPI-test (°)**	69.9 ± 11.2	79.0 ± 13.5	9.1	3.8- 14.5	0.001^†^

SD = Standard Deviation, NDT = Navicular Drop Test, NDT Stance = Navicular Drop Test Stance, NDT SL-S = Navicular Drop Test Single Limb-Stance, AJD = Ankle Joint Dorsiflexion, MTP I = First Metatarsophalangeal Joint, CI = Confidence Interval, † = significant difference between males and females (p < 0.05).

## Discussion

This study showed a good reproducibility of the AJD-test, with an ICC of > 0.85 for the reliability and a small range of 95% LOAs, indicating a good agreement. However, the reproducibility of the NDT and extension MTP1-test was moderate to low. Furthermore, a difference was found between females and males for the NDT (Stance and Single Limb-Stance) and the extension of the MTP1, but not for the mobility of the AJD.

### Navicular Drop Test (NDT)

Studies have reported NDT Stance values for the intrarater reliability in the range of 0.51 to 0.97 [9,26-28] and interrater reliability of 0.46- 0.95 [9,12,27,28]. While our NDT Stance values were similar, with a smaller SD, the intrarater reliability was lower and the interrater reliability in the same range. The SEM of 2.5 mm for the intrarater agreement in our study is in the range of 0.4- 2.7 mm as reported in the literature [9,12,26,27]. However, the 95% LOA was higher, 11.7 mm, as compared to the study by Evans et al. [12], who found a 95% LOA of 5.2 mm. Our SEM for the interrater agreement (3.2 mm) was higher as reported in the literature with a SEM in the range of 1.4- 2.7 mm [9,27,28]. Also the 95% LOA’s for the interrater agreement were wider than those in the study of Shultz et al. [9] who found values between 1.4 and 2.6 of the 95% LOAs by four testers. In addition, both ICC’s of 0.41 and 0.37 and SEMs of 5 and 2.5 mm for the interrater and intrarater reproducibility of our findings for NDT Single Limb-Stance differ from those of Vinicombe et al. [8], who reported a higher reliability (range of 0.33 to 0.76) and lower SEMs of 1.06 to 1.87 mm. So, our results were disappointing.

The most important factors that influence reliability and agreement are the experience of the examiners [9,12], the consistency of placing the subtalar joint in its neutral position by palpation of the talar head [29-32], and identification of the navicular bony landmark [13]. As we used the strategy (sit-to-stand) of McPoil et al. [13] to ensure a difference in the neutral and resting positions of the talar, we considered it unnecessary to place the subtalar joint in neutral position, by palpating the talar head. However, it is possible that small differences in neutral foot position could explain the lower reproducibility of our measurements compared with those of the literature [8,9,12,27,28]. Sell et al. reported that the subtalar neutral position can be measured reliably by palpating the talar head. This should be included in our protocol, to guarantee uniformity of neutral position of the foot.

Two experienced examiners, who were extensively trained in standardization of the tests, performed the measurements. While the examiners had no difficulty in identifying the navicular bony landmark, they had difficulty locating the navicular tuberosity because of anatomical variation among individuals. In some cases, the medial prominence of the navicular was easily palpated and marked. In other cases, the morphology of this bone made the location of the reference point difficult. The navicular bony landmark was marked on the skin with the runner in sitting position but moving the skin could move the marker. Sell et al. [27] reported ICC values of 0.73– 0.96, with the landmark being identified with subjects in prone position, which may be the most optimal way to identify the navicular bony landmark.

Another explanation for the lower reliability in our study is that we measured the height of the navicular bony landmark with a ruler. The ruler was used so to observed the navicular drop directly and it is less time consuming than using a blank card [8], metrecom [26] or digital images [13]. However, as the ruler is placed at an angle, measurements might differ depending on the angle at which the examiner looks at the ruler. A 1.5 × 3-inch note card, as used by Sell et al. [27], might be better than a ruler. Using digital height gauge in measuring the navicular height for avoiding reading error could be most ideal. Taken together, we conclude that we need to adapt the measurement protocol to increase reproducibility.

### Ankle joint dorsiflexion

We followed the advice of Gatt and Chockalingam [33] to standardize the AJD test for runners. With the running position as basis, four variables were standardized: subject position, foot position, placement of the ankle joint axis, and force on the plantar forefoot. We found that AJD can be reliably measured in a weight-bearing position with the knee extended by experienced examiners using a digital inclinometer in runners. Although the data were for asymptomatic runners, they were comparable to the findings of Munteanu et al. [11]. Munteanu found for the intrarater reliability an ICC of 0.77 with a 95% LOA of -9.1° to 8.3° and an interrater reliability of 0.95 (ICC) and a 95% LOA of -5.7° to 8.3°. Interrater and intrarater reliability was the same in our study, possibly reflecting the experience of the examiners, the efficacy of pre-training, the standardized protocol, and the subjects (healthy runners). We did find a systematic error of 1.3° (p < 0.05) for the intrarater reliability of the AJD test and a SEM “agreement” of 8.7°. When calculating the SEM “consistency” and not taking account the systematic errors [24], we obtained a value of 2.34°. We could not identify the source of this systematic error.

In order to interpret the agreement between and within the examiners, 95% LOAs were calculated [21], to determine to what extend whether a difference in AJD can be attributed to a measurement error. The observed difference should be greater than 6.3° and 7.9° when measurements were performed by the same examiner or different examiners, respectively.

### Extension First Metatarsophalangeal Joint (MTP1)

MTP1 extension was measured in a static weight-bearing position, to simulate the running toe-off. To our knowledge, the reproducibility of this test has not been tested in runners previously. Hopson et al. [10] found, in a cohort of 20 healthy adults subjects, much higher reliability values. In our study, we marked the bony landmarks each time MTP1 extension was measured, whereas Hopson et al. [10] marked the bony landmark once for all measurements which could explain the difference in reliability. Furthermore, Hopson et al. [10] drew lines on the first metatarsal, the estimated joint centre, and on the hallux as reference lines for measurements, whereas we used dots to perform the marking quicker. This may decrease the precision with which the goniometer was placed. Step length was also standardized in the study of Hopson et al. [10]. In our protocol the knee was fully extended and the maximum stress on the MTP1 joint reached without balance problems. The possible difference in the size of the step length was not expected to influence the outcome of the MTP1 mobility.

MTP1 extension values of our study (79.4 ± 10.9°, 74.2 ± 13.1° and 80.5 ± 10.8°) were similar to that reported by Buell et al. [34], namely, 82° on passive extension of MTP1 with measurements being validated by radiography. Hopson et al. [10] found greater angles, probably generated by the differences in how anatomical reference points were marked. The SEM for the interrater agreement of 34.4° is high although in line with the low reproducibility. Probably, it could have been helpful to calculate the intrarater reproducibility of examiner MW as well and so identify possible examiner inconsistencies [20], which could explain the high value of the SEM of 34.4°. However, we decided to provide only the intrarater reliability of examiner HD. We chose this option to limit the time involvement of the participating runners. The total time for the measurements of one runner was about an hour, including the breaks in between. If the other examiner had taken the tests twice for every runner, the randomization schedule had to be adapted and probably runners had to spend more than two hours while being measured. Furthermore, the SEM_consistency,_ which not included the systematic error [24], gave a value of 9.1° and is more in the line with the findings of the intrarater agreement of this study. So, the high SEM_agreement_ (34.4°) of the interrater agreement is possibly based on a systematic error.

### Sex difference

In our study a difference was found between male and female runners for the navicular drop and extension of the MTP1. No difference was found for the AJD between male and female runners.

In the studies of Allen et al. [26] and McKeon et al. [19] no difference was found for the ND between males and females. Allen et al. [26] reported only the ND values for the ACL-injured group (mean ND of 10.2 and 10.7 mm for female and males, respectively) and did the NDT measurement with a metrecom. In the study of McKeon et al. [19] a sex difference of 0.1 was found with a 95% confidence interval (CI) of -0.01 to 0.24 mm in a cohort of 118 healthy adults and used the same protocol (sitting to stand) as in our study. However, McKeon et al. [19] did the seating measurements in subtalar neutral position and measured the navicular drop with a straight edge ruler. This could explain the difference with our findings (mean differences of 1.8 mm and 95% CI of 0.6 to 2.9). But also the difference in age of the study population could explain the difference in findings. McKeon et al. [19] used a greater number of participants (57 male and 61 female volunteers) with a younger age (mean age of 21.1 ± 3.0 years and 20.0 ± 1.6 years for male and female, respectively) than in our study, with 22 males and 20 females runners (mean age of 39.1 ± 14.7 years and 37.2 ± 9.3 years for male and female, respectively). It is possible that with increasing age the difference in ND between male and female runners is increased and this may explain the difference in findings between McKeon et al. [19] and ours. Further research in runners, with a more reliable measurement tool is needed before sex differences in ND, as found in our study, can be used in the theoretical model for explaining the risk profile differences between male and female runners.

We found no other studies in the literature with regards to possible sex differences for the ND Single Limb-Stance, extension of the MTP1 and the AJD.

Caution is needed when using the results of the data of the NDT Single Limb-Stance and extension of the MTP1-test to estimate sex differences because of the low reproducibility of these two tests.

This study has some limitations. First, the standardization of the NDT was not optimal. Concerning the NDT, in our protocol the sitting position was used as neutral position of the foot instead of palpating the talar head, so the ND could be determined directly and the measurement time was minimized. The measurement time of the protocol of the extension of MTP1-test [10] was optimized by refraining from using a tapeline and standardized step length. However, to guarantee standardization, it was ensured that all participants reached the maximal stretch of the MTP joint with an extended knee, so step length did not influence the MTP1 extension. It was deliberately chosen to deviate slightly from the existing protocols in the literature to optimize the performing speed of the tests and to facilitate test performance in practice. Given the fact that we were planning a large epidemiological study on risk factors for running injuries we needed tests that were relatively easy to administer (for logistical reasons).

Secondly, by the possibility of using one set of intra-rater results, the examiner consistency in our study was not optimal to determine. Future studies should include a minimal of two sets of intra-rater results so the degree of examiner consistency can be calculated and discussed.

Furthermore, in the review of Menz [35] was stated that the navicular drop was possibly influenced by foot length. Nielsen et al. [36] found that foot length had a significant influence on the navicular drop in both men and women and that this could have been incorporated in the measurement protocol.

## Conclusion

The reliability of the NDT, AJD and MTP1 extension tests have not yet been established in healthy adult runners, even though this population is of particular interest for screening purposes. This study fulfils this need and demonstrates that AJD can be measured reliably in runners (ICC > 0.85) with good interrater agreement (SEM 2.4°–8.7°). Furthermore, we found no differences in AJD between female and male runners. In contrast, the NDT (both Stance and Single Limb-Stance) and the extension of the MTP1 in weight-bearing position had a moderate and low reliability.

## Competing interests

No sources of funding were used to assist the preparation of this study. The authors have no conflict of interest that is directly relevant to the content of this study.

## Authors’ contributions

All the authors have made substantial contributions to the design of the study, analyses and interpretation of the data and drafting of the manuscript. MW did the data collection and statistical analyses. The material within has not been and will not be submitted for publication elsewhere except as an abstract. All authors read and approved the final manuscript.

## Pre-publication history

The pre-publication history for this paper can be accessed here:

http://www.biomedcentral.com/1471-2474/15/171/prepub

## Supplementary Material

Additional file 1 **Video 1.** Navicular Drop test (NDT). Click here for file

Additional file 2 **Video 2.** Ankle Joint Dorsiflexion test (AJD-test). Click here for file

Additional file 3 **Video 3.** Extension First Metatarsophalangeal Joint test (MTP1-test). Click here for file
